# SOCIAL MEDIA USE, KNOWLEDGE, AND ATTITUDES TOWARDS PEOPLE LIVING WITH HIV/AIDS AMONG HIGH SCHOOL STUDENTS IN AN INDONESIAN TOURISM REGION

**DOI:** 10.21010/Ajidv18i2S.4

**Published:** 2024-07-04

**Authors:** NINDREA Ricvan Dana, DARMA Ika Yulia, SUKMA Muthia, MARDIAH Ainil, AGUSTIAN Dede Rahman, SHOLIHATI Mursyidah

**Affiliations:** 1 Department of Medicine, Faculty of Medicine, Universitas Negeri Padang, Bukittinggi, Indonesia, 26181; 2 Department of Midwifery, Universitas Syedza Saintika, Padang, Indonesia, 25132

**Keywords:** Social media, Knowledge, Attitudes, HIV/AIDS, Students, Tourism regions

## Abstract

**Background:**

Social media has become an integral part of adolescent life in Indonesia, particularly in tourism regions. It serves as a platform for disseminating information, including about HIV/AIDS. However, it also has the potential to spread misinformation and harmful content, which can increase stigma and discrimination against people living with HIV/AIDS (PLWHA).

**Aim:**

The aim of this study was to determine the relationships between social media use, knowledge, and attitudes towards PLWHA among high school students in an Indonesian tourism region.

**Methods:**

This research utilized a school-based cross-sectional study design in several high schools located in Bukittinggi City, a renowned tourist destination in West Sumatra Province, Indonesia. The study sample comprised high school students aged 15-18 years, with a total of 118 respondents selected. The sample was chosen using a multistage stratified clustered sampling method. The variables measured in this study included social media usage, HIV/AIDS knowledge, and attitudes towards PLWHA. To test the research hypotheses, data analysis was conducted using structural equation modeling techniques. P<0.05 is considered significant.

**Results:**

There were relationships between social media use and knowledge of HIV/AIDS (β=0.614, t-value=9.327, p-value=<0.001), knowledge of HIV/AIDS and attitudes towards PLWHA (β=0.601, t-value=8.344, p-value=0.014) and social media use and attitudes towards PLWHA (β=0.218, t-value=2.469, p-value=<0.001).

**Conclusion:**

This study confirmed significant relationships were found between social media use, knowledge, and attitudes towards PLWHA. The results highlight the necessity for comprehensive interventions and ongoing support to promote the well-being of students amid the dynamic changes in global tourism.

## Introduction

HIV/AIDS has become a significant global health concern, and Indonesia has seen an increase in new cases. Adolescents are especially at risk of contracting HIV/AIDS (Mahendradhata *et al.*, 2019; Magnani *et al.*, 2022; Nindrea *et al.*, 2023). In Indonesia, as of 2020, there were 540,000 people living with HIV, with a prevalence rate of 0.4% among adults aged 15–49. Among those affected, the largest proportion of HIV/AIDS cases (59.1%) were high school graduates (Jocelyn *et al.*, 2024).

Social media has become an integral part of adolescent life in Indonesia, particularly in tourism regions. It serves as a platform for disseminating information, including about HIV/AIDS (Ibrahim *et al.*, 2024). However, it also has the potential to spread misinformation and harmful content, which can increase stigma and discrimination against people living with HIV/AIDS (PLWHA) (Iribarren *et al.*, 2018). Adolescents’ knowledge and attitudes towards HIV/AIDS are crucial in preventing its spread (Cao *et al.*, 2017). Those who are well-informed about HIV/AIDS and hold positive attitudes towards people living with the disease are more likely to avoid risky behaviors and offer support to those affected (Elghazaly *et al.*, 2023; Mahat, 2019).

Tourism regions in Indonesia have significant potential for the spread of HIV/AIDS due to high sexual activity in these busy areas (Jocelyn *et al.*, 2024; Mahendradhata *et al.*, 2019). This study aims to explore the role of social media, and the knowledge and attitudes of adolescents towards HIV/AIDS in these regions. Therefore, this research can contribute to efforts to prevent HIV/AIDS among adolescents and raise public awareness of the importance of HIV/AIDS education and campaigns through social media.

## Materials and Methods

### Study design and setting

The study employed a cross-sectional design conducted in September 2023 at several high schools in Bukittinggi City. This city is renowned as a tourist destination in West Sumatra Province, Indonesia.

### Study sample

The study sample comprised high school students aged 15-18 years, whose names were included in the education office registry. The sample size of 118 respondents was determined using the proportion sample formula. Inclusion criteria included being aged 15-18 years, able to read and write in Bahasa Indonesia, while exclusion criteria included being absent or not enrolled in high school at the time of the study. Sample selection utilized multistage stratified clustered sampling.

### Measures

The constructs in this study were defined and measured using dependable and validated scales that were previously introduced in well-regarded peer-reviewed journals. Social media use was assessed through two questions: whether individuals delivered messages related to HIV prevention, and if their posts indicated risk behaviors related to overdose and being in an HIV risk group (such as injection drug use, history of drug overdose, inconsistent condom use, number of sexual partners, and engaging in sex in exchange for drugs) (Ibrahim *et al.*, 2024). Knowledge about HIV refers to an individual’s understanding of basic facts related to HIV/AIDS, including information about transmission, symptoms, prevention, and management of the disease (Elghazaly *et al.*, 2023). Attitudes towards PLWHA were assessed based on students’ attitudes towards facts, stigma, discrimination against vulnerable individuals who have been infected with HIV/AIDS, and their willingness to be active agents in fighting HIV/AIDS (Maehara *et al.*, 2019).

### Data collection

The study involved conducting interviews using a research questionnaire. Prior to the interviews, informed consent was obtained, and students were assured that they could decline participation without facing any consequences within or outside the school. To ensure confidentiality, respondents’ names were not disclosed, and the school’s name was kept anonymous to prevent any potential repercussions.

### Ethical approval

This research had obtained approval from the research ethics committee, Dr. M. Djamil General Hospital, Padang, Indonesia (No. DP.04.03/D.XVI.XI/537/2023).

### Data analysis

In this study, descriptive data were displayed using frequencies, percentages, and median values, analyzed with SPSS version 25.0. SmartPLS was employed to assess the reliability and validity of the measures, including Cronbach’s alpha, composite reliability (CR), and average variance extracted (AVE). Furthermore, structural equation modeling (SEM) techniques were utilized to test the research hypotheses, with significance set at p<0.05.

## Results

Characteristics of respondents (Table 1).

**Table 1:** Characteristics of respondents

Variables	Value (n=118)
**Sex, f(%)**	
Male	46 (39.0)
Female	72 (61.0)
**Age, median (IQR)**	17 (14-18)
**Family income, f(%)**	
< regional minimum wage	86 (72.8)
≥ regional minimum wage	32 (27.2)
**Number of family members, median (IQR)**	5 (2-9)
**Father’s education, f(%)**	
Low (< Senior high school)	44 (37.3)
Moderate (Senior high school)	53 (44.9)
High (University)	21 (17.8)
**Mother’s education, f(%)**	
Low (< Senior high school)	29 (24.6)
Moderate (Senior high school)	51 (43.2)
High (University)	38 (32.2)
**Father’s occupation, f(%)**	
Government employee	9 (7.6)
Private sector employee	6 (5.1)
Entrepreneur	38 (32.2)
Small-scale merchant	17 (14.4)
Farmer	12 (10.2)
Others	36 (30.5)
**Mother’s occupation, f(%)**	
Government employee	18 (15.3)
Private sector employee	6 (5.1)
Entrepreneur	15 (12.7)
Small-scale merchant	5 (4.2)
Farmer	2 (1.7)
Housewife	72 (61.0)

Table 1 shows that the majority of respondents were female (61.0%) and male (39.0%). The median age of the respondents was 17 years. More than half of the respondents had a family income less than the regional minimum wage (72.8%). The median number of family members was 5. The most common education level for both fathers and mothers was moderate. The most common occupation for fathers was entrepreneur (32.2%), and for mothers, it was housewife (61.0%).

Results of confirmatory factor analysis (Table 2).

**Table 2:** Results of confirmatory factor analysis

Constructs/ factors	Standardized factor loadings
*Social media use (α =0.845; CR=0.856; AVE=0.662)*	
SM1 - Deliver messages related to HIV prevention	0.742
SM2 - Posts indicated risk behaviors related to overdose and being in an HIV risk group (such as injection drug use, history of drug overdose, inconsistent condom use, number of sexual partners, and engaging in sex in exchange for drugs)	0.731
*Knowledge of HIV/AIDS (α =0.872; CR=0.832; AVE=0.676)*	
K1- HIV is contagious	0.812
K2 - HIV does not get transmitted through daily contact and using public bathrooms	0.853
K3 - HIV does not transmit by contacting saliva, tears, and sweat	0.871
K4 - HIV does not transmit through coughing and sneezing	0.778
K5 - HIV is transmitted by blood transfusion	0.780
K6 - HIV is sexually transmitted	0.801
K7 - HIV-infected person may look healthy and feel healthy	0.880
K8 - HIV-infected individuals develop signs of the infection quickly	0.778
K9 - HIV patients can transmit the virus at any disease stage	0.866
K10 - HIV is not curable	0.823
*Attitudes towards PLWHA* *(α =0.889; CR=0.853; AVE=0.678)*	
A1 - I do not want to give salaam when greeting someone who is HIV/AIDS positive	0.774
A2 - Adolescents who are HIV-positive should be banned from attending schools with HIV-negative students	0.784
A3 - Adolescents who are HIV-positive should not swim in a pool with HIVnegative students	0.811

α = Cronbach’s alpha; CR, composite reliability; AVE, average variance extracted

Table 2 indicated that all standardized factor loadings were > 0.60 and statistically significant (p<0.05). The reliability of the variables was assessed using Cronbach’s α values, all of which exceeded ≥ 0.70, indicating that the variables were reliable. To gauge the convergent validity of the scales, we considered three criteria. Firstly, all indicator loadings were > 0.70. Secondly, the composite reliabilities (CR) surpassed 0.8. Thirdly, the average variance extracted (AVE) for each construct ≥ 0.5. These results confirmed satisfactory convergent validity for the research constructs.

Results of discriminant validity (Table 3).

**Table 3:** Results of discriminant validity

No	Constructs	1	2	3
1	Social media use	*0.723*		
2	Knowledge of HIV/AIDS	0.261	*0.736*	
3	Attitudes towards PLWHA	0.322	0.295	*0.742*

Italicized values on the diagonal indicate the AVE, while the remaining values represent the squared interconstruct correlations.

Table 3 assessed discriminant validity, which measures the extent to which measures of three variables were empirically distinct. The AVE of each latent construct had to be greater than the squared correlations between that construct and the rest of the latent constructs. This analysis demonstrated that these conditions for discriminant validity were fulfilled.

**Figure 1:**
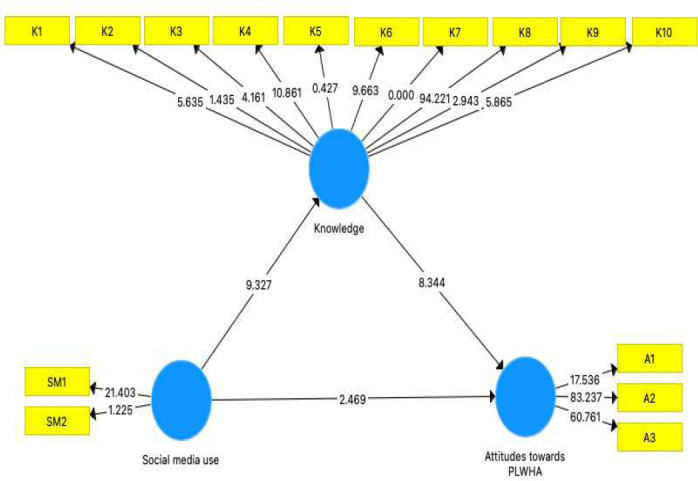
Findings regarding the hypothesized relationships

Findings regarding the hypothesized relationships (Table 4 and Figure 1).

**Table 4:** Results of the relationships of social media use, knowledge, and attitudes towards PLWHA among high school students in an Indonesian tourism region

Path specified	Coefficient (β)	t-value	p-value	Result
Social media use -> Knowledge of HIV/AIDS	0.614	9.327	<0.001	Accepted
Knowledge of HIV/AIDS -> Attitudes towards PLWHA	0.601	8.344	0.014	Accepted
Social media use -> Attitudes towards PLWHA	0.218	2.469	<0.001	Accepted

Table 4 and Figure 1 illustrate that there were relationships between social media use and knowledge of HIV/AIDS (β=0.614, t-value=9.327, p-value=<0.001), knowledge of HIV/AIDS and attitudes towards PLWHA (β=0.601, t-value=8.344, p-value=0.014) and social media use and attitudes towards PLWHA (β=0.218, t-value=2.469, p-value=<0.001).

## Discussion

The results of this study showed a significant relationship between social media use, knowledge about HIV/AIDS, and attitudes towards PLWHA among high school students in the tourist area of Bukittinggi. This reflected how social media could function as an effective educational tool in enhancing understanding of HIV/AIDS (Aghaei *et al.*, 2023). On the other hand, this phenomenon also underscored the importance of quality control over the information disseminated through social media platforms to ensure that the information received by teenagers was accurate and did not add to the stigma against PLWHA (Yu *et al.*, 2023).

Previous study had shown that social media use could influence teenagers’ knowledge and attitudes towards HIV/AIDS (Ibrahim *et al.*, 2024). Several studies found that social media could be a significant source of health information, including about HIV/AIDS (Magnani *et al.*, 2022; Lipoeto *et al.*, 2020; Nindrea *et al.*, 2018). Good knowledge about HIV/AIDS was crucial for forming positive attitudes and reducing stigma towards PLWHA (Iribarren *et al.*, 2018; Harahap *et al.*, 2017). However, previous research also indicated that misinformation or stigma spread on social media could influence negative attitudes towards PLWHA (Cao *et al.*, 2017).

The strength of this study was that it was the first to investigate social media use, knowledge, and attitudes towards PLWHA among high school students in an Indonesian tourism region. However, this study had several limitations. First, the cross-sectional design limited the ability to draw causal conclusions. Second, the study was conducted in only one tourist city in Indonesia, so the results might not be generalizable to other areas with different characteristics. Third, the measurement of variables was based on self-reports, which could be prone to respondent bias. Lastly, social media use was measured in general terms without considering the specific types or content consumed by the respondents.

Based on the findings of this study, it is recommended to develop health education programs that utilize social media more effectively to deliver accurate information about HIV/AIDS. Additionally, further research with a longitudinal design is needed to confirm the causal relationships between social media use, knowledge, and attitudes towards PLWHA. Studies conducted in various regions with different demographic and social characteristics are also necessary to broaden the generalizability of the findings. Finally, it is important to develop strategies to minimize bias in self-reports by integrating additional measurement methods such as observations or in-depth interviews.

## Conclusion

In conclusion, this study provides valuable insights into the relationships among social media use, knowledge, and attitudes towards PLWHA among high school students in an Indonesian tourism region. The findings underscore the need for holistic interventions and continuous support to ensure the well-being of students in the evolving landscape of global tourism.
